# Shared Extended-Spectrum β-Lactamase-Producing *Salmonella* Serovars between Agricultural and Aquatic Environments Revealed through *invA* Amplicon Sequencing

**DOI:** 10.3390/microorganisms8121898

**Published:** 2020-11-30

**Authors:** Cecilia Mahlatse Raseala, Mutshiene Deogratias Ekwanzala, Maggy Ndombo Benteke Momba

**Affiliations:** Department of Environmental, Water and Earth Sciences, Arcadia Campus, Tshwane University of Technology, Private BagX680, Pretoria 0001, South Africa; ceciliamahlatse5@gmail.com (C.M.R.); ekwanzala.md@gmail.com (M.D.E.)

**Keywords:** extended-spectrum beta-lactamases, *Salmonella* spp., agricultural environment, water

## Abstract

The presence of antibiotic-resistant *Salmonella* spp. in the environment is of great public health interest, worldwide. Furthermore, its extended-spectrum β-lactamase (ESBL)-producing strains constitute an emerging global health concern due to their limited treatment options in hospital. Therefore, this study aimed at characterising and tracking nonresistant and ESBL–producing *Salmonella* spp. from agricultural settings to nearby water sources highlighting their antibiotic resistance genes (ARG) and virulence factor (VF) distribution using a combination of both culture-dependent and independent methods. Furthermore, this study investigated the diversity and shared serovars among sampled matrices using amplicon sequencing of the invasion gene A (*invA*) of *Salmonella* spp. The results showed that soil had the highest prevalence of *Salmonella* spp. (62.5%, 65/104) and ESBL-producing *Salmonella* (34.6%, 36/104). For typed ARG, the most commonly detected gene was *bla*_OXA_ with 75% (30/40), followed by *bla*_CTX-M_ 67.5% (27/40)_,_ *bla*_TEM_ 40% (16/40) and *sul*1 30% (12/40) gene; *bla*_SHV_ gene was not detected in isolated ESBL-producing *Salmonella* spp. For VF, the most detected gene was *invA* (96.9%, 38/40), followed by *spa*M (17.5%, 7/40), *spi*C (40%, 16/40), *orf*L (32.5%, 13/40), *mis*L 32.5% (13/40) and *pip*D 32.5 (13/40). For diversity analysis, soil, manure, irrigation water and nearby freshwater revealed 81, 68, 12 and 9 serovars, respectively. Soil, manure, irrigation water and freshwater stream samples shared five serovars, which indicated circulation of ESBL-producing *Salmonella* spp. within the agricultural environment and nearby water sources. Soil is therefore identified as one of the major reservoirs of ESBL-producing *Salmonella* spp. It is concluded that agricultural environment contamination may have a direct relationship with the presence of antibiotic-producing *Salmonella* in freshwater streams.

## 1. Introduction

*Salmonella* spp. are a member of the *Enterobacteriaceae* family, and consist of two main species, namely *S. enterica* and *S. bongori*. Of the two species, *S. enterica,* has been reported to comprise more than 2500 serovars [1], which are separated based on their differences in lipopolysaccharide layer with regard to their somatic (O) and flagellar (H) antigens [2]. These serovars are the most critical foodborne and waterborne pathogens, causing thousands of hospitalisations and deaths worldwide. According to Stanaway et al. [3], nontyphoidal *Salmonella* spp. was linked to 535,000 infection cases worldwide with the highest incidence from subSaharan Africa (34.5%) and children under the age of five (34.3%) were the most affected. In the United States, for example, *Salmonella* spp. outbreak was reported in 40 states where the consumption of imported Mexican cucumbers caused illness in at least 907 people, with six deaths from July 2015 to February 2016 [4]. In South Africa, the Enteric Diseases Centre of the National Institute for Communicable Diseases reported a *Salmonella* spp. outbreak with approximately 4966 cases associated with food- and waterborne diseases between 2003 and 2005 [5].

It has been recorded that *Salmonella* spp. can be transmitted through water, food, soil or person-to-person contact [1]. Nevertheless, contaminated fresh produce and raw meat are well-known as the primary vehicles for *Salmonella* spp. transmission to humans [6,7,8]. This pathogen can also be spread through the faecal-oral route of contamination where the animal or human faeces enter the aquatic environment, such as rivers, directly from agricultural land through runoff [9]. It has been reported that *Salmonella* spp. can persist for an extended time in soil, water and faeces excreted in the environment [1]. This situation is particularly a matter of concern in low- and middle-income countries (LMICs), where surface water is used for domestic purposes. Poor water quality has continuously led to the world burden of waterborne infections [10]. Furthermore, poor sanitation and inadequate potable water supply make contributions to the increase in enteric fever and diarrhoea [11], leading to an everlasting prevalence of water-related infectious agents attributed to this water source. This situation is amplified by the recent emergence of antibiotic-resistant *Salmonella* spp. strains.

The development of antibiotic resistance (AR) is thought to arise from the release of antibiotics into environmental settings [12] such as agricultural and farming environments. It has been reported that approximately 70% of antibiotics produced are used for animal prophylaxis and as growth promoters [13]. This overuse and misuse of antibiotics in animal farming have impacted the quality of animal manure applied to agricultural soil. Moreover, there is an increasing reliance on wastewater for irrigation, which also disperses resistant bacteria from wastewater to nearby freshwater [14].

*Salmonella* spp., like other bacteria in the *Enterobacteriaceae* family, have become resistant to antibiotics previously effective in treating the same infections. The emergence of multidrug-resistant *Salmonella* spp. has been reported worldwide [15]. Parisi et al. [15] reported that multidrug serovars are mostly related to Typhimurium, Enteritidis, Newport and Heidelberg. Furthermore, infections brought by AR *Salmonella* spp. have been reported to be associated with excess bloodstream infections and higher mortality compared to their pansusceptible strains [15]. Of these resistant strains, there are extended-spectrum β-lactamases (ESBL)-producing *Salmonella* spp. In 2017, ESBL-producing *Enterobacteriaceae* family, which *Salmonella* spp. belong to, was set as a high priority organism for research and new antibiotic development [16]. A more severe case of ESBL called carbapenem resistance is thought to have developed during ertapenem treatment of ceftriaxone-resistant and ciprofloxacin-resistant *Salmonella enterica* serotype Typhimurium [17]. The emergence and spread of ESBL production among isolates of *Enterobacteriaceae*, both from community and healthcare settings, have engendered fear. Several β-lactamase enzymes such as TEM, SHV, CTX-M and OXA have been observed in *Salmonella* serovars from all over the world. Extended-spectrum β-lactamases have negatively impacted clinical outcomes, resulting in expensive treatment options [18]. Fewer strategies have been proposed to address the contributions of animal and environmental reservoirs to the dissemination of antibiotic resistance in LMICs. 

Surveillance and serotyping of *Salmonella* have been conducted worldwide [19], and several studies carried out have documented the direct transfer of antibiotic-resistant bacteria from animals to humans. Molecular techniques such as polymerase chain reaction (PCR), restriction fragment length polymorphism (RFLP), sequencing of H antigen genes (*fliC* and *fljB*), multiplex PCR of the O-antigen encoding genes (*tyv* and *wzx*), phylogenetic analyses of the beta subunit of ATP synthase gene (*atpD*) and automated ribotyping system, 16S–23S rRNA spacer have been developed for typing *Salmonella* serovars. However, their discriminative ability is insufficient for practical applications [19]. Most studies have reported the dissemination of ESBL-producing *Salmonella* spp. from agricultural environments [20,21,22], and only a few of them have focussed on ESBL-producing *Salmonella* spp. Limited investigation on ESBL production in agricultural environments could be due to low concentrations of ESBL-producing bacteria in these environments compared to clinical settings.

Consequently, this study aimed at characterising and tracking the prevalence of *Salmonella* spp. versus ESBL-producing *Salmonella* spp. in agricultural and aquatic environments as well as their antibiotic resistance genes and virulence gene distribution. Furthermore, this study investigated the diversity and shared serovars among sampled matrices using amplicon sequencing of the *invA* gene of *Salmonella* spp.

## 2. Materials and Methods

### 2.1. Study Site Description and Sample Collection

The study site and sample collection are described in detail by Raseala et al. [23].

### 2.2. Media and Sample Preparation

All media were prepared according to the manufacturers’ instructions. CHROMagar™ Salmonella Plus media and CHROMagar™ Salmonella Plus media supplemented with ESBL supplement (Media Mage, Johannesburg, South Africa) were used for the isolation of *Salmonella* spp. and ESBL-producing *Salmonella* spp., respectively.

Sample collection and preparation was performed as described by Raseala et al. [23].

### 2.3. Detection and Isolation of ESBL-Producing Salmonella spp. and Salmonella spp.

For the detection and isolation of *Salmonella*, a loopful of the overnight suspension in tryptic soy broth was streaked using a streaking loop on ESBL-supplemented and unsupplemented CHROMagar™ Salmonella Plus media to detect and isolate non-ESBL-producing *Salmonella* spp. and ESBL- producing *Salmonella* spp., respectively. The positive controls *Salmonella* Typhimurium ATCC 14028 (ThermoFisher Scientific, Johannesburg, South Africa) and *Salmonella enterica* subsp. *enterica* serovar Typhimurium strain ATCC 13311 (ThermoFisher Scientific) were used as quality control cultures for the isolation of *Salmonella* spp. and ESBL-producing *Salmonella* isolates. The plates were incubated at 37 °C for 18 to 24 h, and samples were analysed in triplicate.

To obtain pure cultures for serotyping, antibiotic-resistant gene (ARG) and virulence factor (VF) detection, typical growth colonies on Petri dishes were randomly selected and subcultured again on CHROMagar™ Salmonella Plus media supplemented with ESBL supplement and incubated at 37 °C for 18 to 24 h. Furthermore, the grown colonies were streaked on tryptic soy agar (Sigma-Aldrich, Johannesburg, South Africa) and incubated for 24 h at 37 °C. For *invA* amplicon analysis, all typical colonies of ESBL-producing *Salmonella* spp. were collected, transferred to sterile microcentrifuge tubes, and preserved in 15% glycerol at −20 °C until further analyses. 

### 2.4. Bacterial Confirmation and Identification

To confirm and identify the presumptive isolated ESBL-producing *Salmonella* spp., immunological lateral flow test Singlepath^®^ Salmonella (Merck, Johannesburg, South Africa) test kit and matrix-assisted laser desorption/ionisation-time of flight mass spectrometry (Bruker Daltonik MALDI Biotyper, Billerica, MA, USA) analysis were performed as described by Raseala et al. [23], respectively. Only ESBL-producing *Salmonella* spp. isolates that showed positive reactions on the Singlepath^®^ Salmonella kit were further sent for identification at the MALDI-TOF diagnostic service of the University of Pretoria for identification using MALDI-TOF MS. Data acquisition from the machine was acquired through the MBT Explorer Software plus MBT Compass Library.

### 2.5. Serogrouping of ESBL-Producing Salmonella spp. Isolates

The identified ESBL-producing *Salmonella* spp. were serogrouped using a Wellcolex colour *Salmonella* test kit (Thermo Scientific) following the manufacturer’s instructions. Positive controls with the positive control reagents (green, blue and red controls) were carried out alongside the latex reagents 1 and 2 separately without inoculums. Results were interpreted according to the manufacturer’s guidelines.

### 2.6. DNA Extraction

For the molecular study, the genomic DNA from ESBL-producing *Salmonella* spp. was extracted using the InstaGene™ matrix (Bio-Rad, Johannesburg, South Africa) according to the manufacturer’s instructions as detailed by Raseala et al. [23].

For the *invA* amplicon sequencing analysis, all suspected ESBL-producing *Salmonella* spp. isolates grown on ESBL-supplemented CHROMagar™ Salmonella Plus plates were pooled together per sample source and transferred into a DNase-free Eppendorf tube. These bacterial cells were disrupted in a 2 mL microfuge tube containing 1.5 mL of 1 × PBS and 2% Tween 20 (Sigma Aldrich, South Africa) using a Disruptor Genie^®^ Vortex mixer (Scientific Industries Inc., NY, USA). Each microfuge tube containing bacterial cells was placed under centrifugation at 10,000 × rpm for 5 min, and the resulting bacterial pellet was used to extract genomic DNA using a ZymoBIOMICS™ DNA Miniprep Kit (Zymo Research, Irvine, CA, USA) according to the manufacturer’s instructions.

### 2.7. Detection of ARG in ESBL-Producing Salmonella spp. Using PCR

Five different ARG, namely *bla*_CTX_, *bla*_TEM_, *bla*_OXA_, *bla*_SHV_ and *sul*1, were assessed in 40 randomly selected ESBL-producing *Salmonella* spp. isolates (10 from each matrix). The selected ARG were amplified by PCR using the primers listed in Table 1. Each reaction mixture consisted of 10 mL of SsoAdvanced Evergreen Supermix, 2 µL of each primer (reverse and forward), 5 µL of the DNA template and 3 µL of nuclease-free water, resulting in a final volume of 20 µL per reaction. The primers were prepared according to the manufacturer’s instructions (Inqaba Biotec, Pretoria, South Africa) to obtain stocks at working concentrations of each of the PCR primer. The CFX96 Touch™ real-time PCR detection system (Bio-Rad, South Africa) was used for PCR assays using the following conditions: initial denaturation at 98 °C for 2 min, followed by 40 cycles of amplification of denaturation 98 °C for 5 s, annealing for 30 s (60 °C for *bla*_CTX_, 50 °C for *bla*_SHV_, 58 °C for *bla*_OXA_, 53 °C for *bla*_TEM_ and 65 °C for *sul*1 genes) and a primer extension at 72 °C for 5 s. Bio-Rad CFX Manager software (ver. 3.0) was used to acquire the generated data. The amplicon sizes were checked by running the amplicons on a 1% agarose gel (ThermoFisher, South Africa) stained with ethidium bromide (ThermoFisher, South Africa) and then visualised under a UV transilluminator (InGenius Bio Imaging System, Syngene, Cambridge, UK).

### 2.8. Detection of VF in ESBL-Producing Salmonella spp. Using PCR

Polymerase chain reactions were performed on the same 40 DNA extracts selected for detection of ARG and analysed for five virulence genes (*spa*M, *orf*L, *spi*C, *mis*L and *pip*D). The PCR primers used to amplify internal fragments from the genes mentioned above are shown in Table 1. Amplifications were carried out using the same reaction mixture used to screen ARG. The hot start technique used to prevent nonspecific amplification of the virulence genes was as follows: initial enzyme activation at 98 °C for 2 min, followed by 40 amplification cycles of denaturation at 98 °C for 5 s, annealing (*spi*C gene at 54 °C, *mi*sL and *orf*L at 58 °C, *spa*M 55 °C and *pip*D gene at 55 °C) and a final extension at 72 °C for 6 min.

For the amplicon sequencing analysis targeting the *invA* gene, the CFX96^TM^ Real-time PCR Detection System (Bio-Rad, South Africa) was used for PCR assays. The following cycling parameters were used: initial denaturation at 98 °C for 2 min, following 40 cycles of amplification, denaturation at 98 °C for 5 s, annealing at 58 °C each for 30 s, extension at 72 °C for 1 min and a single final extension at 72 °C for 2 min. The amplicon sizes were checked by running the samples on a 1% agarose gel (ThermoFisher, South Africa) stained with ethidium bromide (ThermoFisher, South Africa) and then visualised under a UV transilluminator (InGenius Bio Imaging System, Syngene, Cambridge, UK). The digital image of the band patterns was acquired and viewed with UV light to determine the presence of the PCR products. The PCR products were sent for next-generation sequencing at Inqaba Biotechnology Industries (Pretoria, South Africa).

### 2.9. InvA Amplicon Sequencing and Analysis

The *invA* gene has been widely used to reveal *Salmonella* spp. diversity in numerous studies [29,30] with the ability to show up to 86 serovars [31]. Amplicon sequencing was carried out on *inv*A gene PCR products at the sequencing centre using an Illumina MiSeq device (Illumina Inc., San Diego, CA, USA), following the manufacturer’s instructions.

### 2.10. Bioinformatic Analysis of the invA Sequences

Quality control and improvements of raw sequences from the sequencer were performed in Galaxy (usegalaxy.org). Briefly, raw reads were imported into the galaxy server. Since paired-end reads were generated, each matrix (irrigation water, soil, manure and nearby freshwater stream) consisted of two separate fastq files, one containing the forward reads, and the other the reverse reads. Using the FASTQ join v.1.1.2-801.1 function [32] in Galaxy, the forward and reverse read generated eight joined samples, namely IW1, IW2, S1, S2, M1, M2, WB1 and WB2. The quality of generated files was assessed using FastQC v.0.72+galaxy1 software [33]. To obtain high-quality sequences for downstream analysis, trimming of low-quality bases and removal of adapters were performed using Trimmomatic v.0.38.0 [34], PRINSEQ v.0.20.4 [35], Trim Galore! v.0.6.3 [36], Fastp v.0.19.5+galaxy1 [37]. All pipelines were run in default settings as set in the Galaxy server. Chimaera removal was performed using UCHIME, according to the de novo method [38]. Removal of human DNA contamination was performed using DeconSeq v.0.4.3 [39]. Generated high-quality sequence reads were then submitted to the Kaiju online web server [40] to obtain the *Salmonella* spp. serovars hit using RefSeq Genomes (proteins from completely assembled RefSeq genomes—bacteria, archaea and viruses) as the reference database (defaults parameters of ticked SEG filter, the Run mode in Greedy (minimum match score of 75 and 5 allowed mismatches) with a minimum match length of 11).

### 2.11. Data Analysis

Matrix prevalence was expressed as the percentage of positive samples over the total number of samples tested. Differences in the prevalence of ESBL-producing *Salmonella* between the four matrices were assessed using the chi-square test (*χ*^2^ test). *Salmonella* spp. serovars richness and Shannon-Wiener (H’) diversities in samples were determined using the vegan R package in Microsoft R Open 3.3.2. One-way ANOVA with a Tukey’s HSD post-hoc test was performed to reveal significant differences among diversity mean across assessed matrices. The analysis was performed at a 95% confidence limit (*p* ≤ 0.05). Graphs were constructed using the ggplot2 package in Microsoft R Open 3.3.2.

## 3. Results

### 3.1. Prevalence of Nonresistant and ESBL-Producing Salmonella spp.

**Figure 1:**
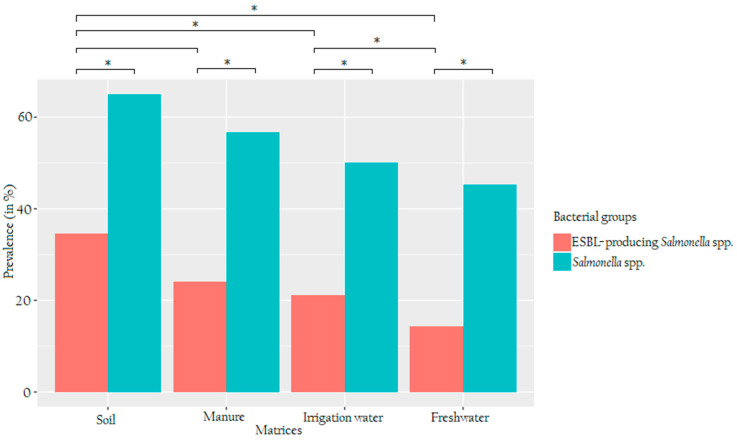
A clustered barplot showing the prevalence of presumptive total and extended-spectrum β-lactamase (ESBL)-producing *Salmonella* spp. The red bars represent the prevalence of ESBL-producing *Salmonella* spp., while the turquoise bars represent total *Salmonella* spp. from different assessed matrices. The X-axis shows the four sampled environmental matrices, whereas the Y-axis shows the prevalence (in %) of total and ESBL-producing *Salmonella* spp. The asterisks denote significant differences between bacterial groups.

In total, 104 samples were collected over thirteen weeks, starting from June 2018 to September 2018, which consisted of 26 individual samples for each matrix (soil, manure, irrigation water and freshwater stream). Once transported to the laboratory, each sample was subdivided into four technical replicates before analysis, making a total of 416 technical replicates to avoid errors. Of the 416 samples, 53.6% (223/416) samples produced presumptive colonies on unsupplemented CHROMagar™ Salmonella Plus media, whereas ESBL-supplemented CHROMagar™ Salmonella Plus media showed a 23.6% (98/416) of the presumptive ESBL-producing *Salmonella* spp. The prevalence of each positive sample for *Salmonella* spp. versus ESBL-producing *Salmonella* spp. per matrix is shown in Figure 1. Overall, both agricultural settings and nearby freshwater harboured *Salmonella* spp. and ESBL-producing *Salmonella* spp. The highest number of presumptive *Salmonella* spp. was found in soil with 62.5% (65/104), followed by manure with 56.731% (59/104), irrigation water 50% (52/104) and lastly freshwater with 45.192% (47/104). In terms of ESBL-producing *Salmonella* spp., the highest incidence of presumptive ESBL-producing *Salmonella* was also found in soil samples with 34.615% (36/104), followed by manure samples 24.038% (25/104) and irrigation water 21.153% (22/104) and the lowest was found in freshwater stream samples with 14.423% (15/104). Statistically, *p*-values through the *χ*^2^ test revealed a significant difference (*p* ≤ 0.05) between total *Salmonella* spp. and ESBL-producing *Salmonella* of the same matrix. Furthermore, *p*-values of ESBL-producing prevalence between was significantly different (*p* ≤ 0.05) for the following matrices soil-manure, soil-irrigation water, soil-nearby water, manure-nearby water and irrigation-water-nearby water, However, no statistically different prevalence were observed for ESBL-producing *Salmonella* spp. between manure-irrigation water (*p* = 0.224).

### 3.2. Bacterial Confirmation and Identification

Only ESBL-producing *Salmonella* isolates that showed a positive reaction on the immunological lateral flow test Singlepath^®^ Salmonella test kit were sent for identification by the MALDI-TOF Biotyper. Out of 147 presumptive ESBL-producing *Salmonella* spp. isolated from all matrices, 134 (91.2%) isolates were identified as *Salmonella* spp. Soil displayed the highest number of isolates identified as *Salmonella* spp. [47.0% (63/134)], followed by manure [30.4% (48/134)], irrigation water [9.7% (13/134)] and lastly nearby freshwater stream with 7.5% (10/134). The remaining identified bacterial species included *Escherichia coli* (5), *Pseudomonas putida* (3), *Pseudomonas monteilii* (2), *Pseudomonas fulva* (1) and *Stenotrophomonas* *maltophilia* (*Pseudomonas hibiscicola*) (2).

### 3.3. Serotyping of ESBL-Producing Salmonella spp. Isolates

Out of 134 [soil (*n* = 63), manure (*n* = 48), irrigation water (*n* = 13) and freshwater stream (*n* = 10)] confirmed ESBL-producing *Salmonella* spp. isolates, 129 isolates were successfully serogrouped, representing all available serogroup types (A, B, C, D, Vi-antigen and E or G serogroups). The highest number of isolated ESBL-producing *Salmonella* spp. belonged to serogroup C, while the lowest was Vi-antigen, with one occurrence in irrigation water. The serogroup A was found in soil (*n* = 12), manure (*n* = 0), irrigation water (*n* = 1) and freshwater stream (*n* = 3). The serogroup B was found in soil (*n* = 15), manure (*n* = 14), irrigation water (*n* = 11) and freshwater stream (*n* = 0). Serogroup C was found in soil (*n* = 21), manure (*n* = 18), irrigation water (*n* = 0) and freshwater stream (*n* = 5). Serogroup D was found in soil (*n* = 13), manure (*n* = 3), irrigation water (*n* = 1) and freshwater stream (*n* = 2). Serogroup E or G was found in soil (*n* = 1), manure (*n* = 9), irrigation water (*n* = 0) and freshwater stream (*n* = 0).

### 3.4. Detection of ARG in ESBL-Producing Salmonella spp.

**Figure 2:**
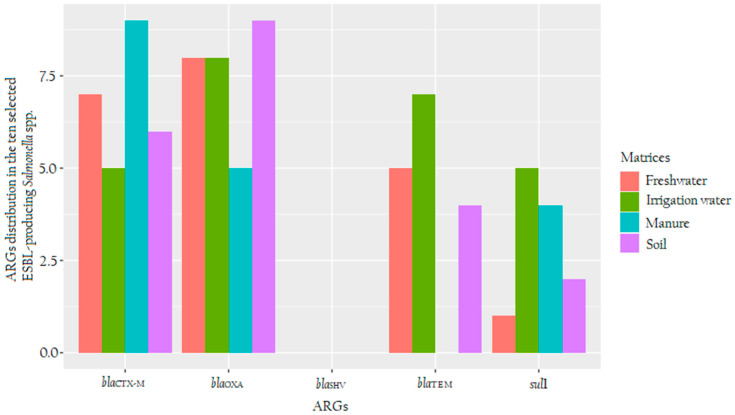
A clustered barplot showing the distribution of antibiotic-resistant genes (ARGs) detected in the ten randomly selected ESBL-producing *Salmonella* spp. from soil (purple), manure (turquoise), irrigation water (green) and nearby freshwater (red). The X-axis shows the five targeted ARGs, whereas the Y-axis shows the number of ARGs found in each assessed matrix.

The distribution of selected ARG in ESBL-producing *Salmonella* spp. assessed in this study is illustrated in Figure 2. Overall, the most commonly detected ARG was *bla*_OXA_ [75% (30/40)] in ESBL-producing *Salmonella* spp. This ARG was followed by 67.5% (27/40) of *bla*_CTX-M_, 40% (16/40) of *bla*_TEM_ and 30% (12/40) *sul*1 genes. Nine ESBL-producing *Salmonella* spp. isolates from soil and manure carried both *bla*_CTX-M_ and *bla*_OXA_, whereas isolates from both irrigation water and nearby freshwater streams displayed each eight detections for *bla*_OXA_. No isolate carried *bla*_SHV_ from all of the assessed matrices.

### 3.5. Detection of VF in ESBL-Producing Salmonella spp.

**Figure 3:**
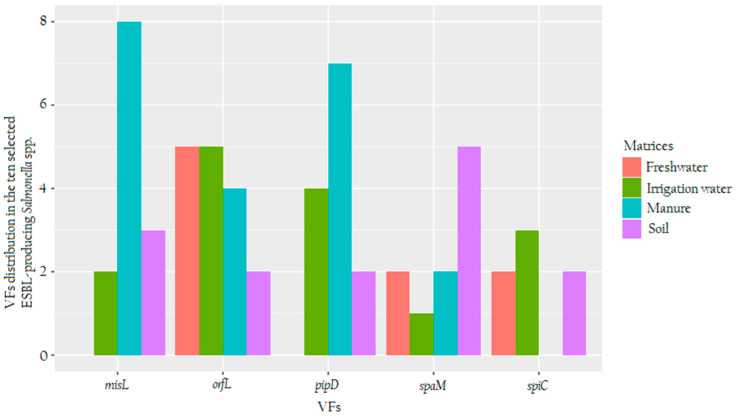
A clustered barplot showing the distribution of virulence factors (VFs) detected in the ten randomly selected ESBL-producing *Salmonella* spp. from soil (purple), manure (turquoise), irrigation water (green) and nearby freshwater (red). The X-axis shows the five targeted VFs, whereas the Y-axis shows the number of ARGs found in each assessed matrix.

The prevalence of VF in the 40 randomly (10 for each matrix) selected ESBL-resistant *Salmonella* spp. isolates is displayed in Figure 3. Among the five VF, only *spaM* and *orfL* were commonly detected in isolates that originated from agricultural settings and the nearby water source. The VF *spaM in* ESBL-resistant *Salmonella* spp. isolates from soil was detected at a rate of 50% and in irrigation water, the rate was 10%, while manure and nearby water displayed a similar rate of 20%. For *orfL,* ESBL-producing *Salmonella* spp. isolates, which originated from irrigation water and nearby water sources harboured a similar rate of 50% and those from manure and soil exhibited rates of 40% and 20%, respectively. 

Two VF—*misL* and *pipD*—were detected in ESBL-producing *Salmonella* spp. isolates from all selected the selected matrices, except for nearby water sources. The highest rate of *misL* was found in isolates from manure (80%), followed by those from soil (30) and irrigation water (20%). *PipD* VF was detected in isolates from manure at a rate of 70%, from irrigation water and soil at rates of 40% and 20%, respectively. The ESBL-resistant *Salmonella* spp. isolates from soil and manure were found to carry *spiC* VF at a similar rate of 5% and those from irrigation water at a rate of 7.5%, while this VF was not detected in manure water. Overall, soil and irrigation water were the only agricultural setting that had ESBL-producing *Salmonella* spp. isolates that harboured all five VF in this study. Manure and nearby water strains harboured four and three VFs (Figure 3), respectively.

### 3.6. InvA Amplicon Analysis

Eight pooled samples per matrix were sequenced using an Illumina MiSeq platform that generated more than 99.8 Mb of unzipped data for processing using Kaiju for taxonomic identification at serovars level.

**Figure 4:**
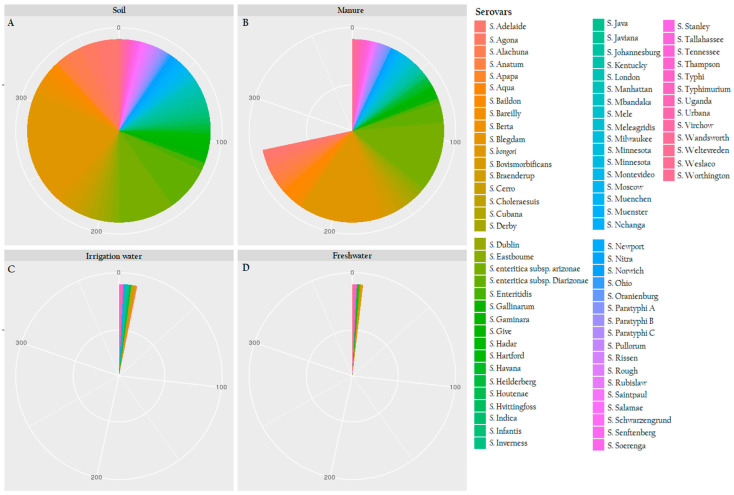
Pie charts showing diversity analysis of ESBL-producing *Salmonella* spp. from (**A**) soil matrix with 81 serovars, and dominant serovars were *Salmonella enterica* subsp. *enterica* serovar Heidelberg with 17%, followed by 11% of *S. enterica* subsp. *enterica* serovar Enteritidis, 8% Newport, 7% Agona, 4% Typhimurium, 3% Montevideo and 1%–2% of other serovars; (**B**) manure matrix with 68 serovars, and dominant serovars were *S.* Heidelberg with 18%, followed by 11% Enteritidis, 10% Newport, 5% Agona, 4% Typhimurium, 3% Senftenberg and 1%–2% of other serovars; (**C**) irrigation water with 12 serovars, and dominant serovars were 17% of *S.* Typhimurium, followed by 11% of *S.* Johannesburg and *S.* Heidelburg; and (**D**) freshwater stream with 9 serovars and the most abundant was *Salmonella* enterica subsp. enterica (19%).

Several subspecies and serovars were assigned as *Salmonella enterica*. Overall, the two species of *Salmonella,* which are *S. enterica* and *S. bongori*, were found with enterica subspecies and diverse serovars. The results showed that the most abundant serovar was *S.* enterica subsp. *enterica* serovar Heidelberg (*n* = 136), followed by *S.* enterica subsp. *enterica* serovar Enteritidis (*n* = 86), Newport (*n* = 68), Agona (*n* = 46), Typhimurium (*n* = 34) and Montevideo (*n* = 20) (Figure 4). 

To support Figure 4 and statistically approve the differences observed, serovar richness and Shannon-Wiener (H’) tests were conducted. The serovar richness test revealed that the soil matrix was the richest (81 serovars), followed by manure (61 serovars) and irrigation water (12 serovars) and the least rich was nearby water sources (nine serovars). The significant differences assessed using the one-way ANOVA revealed a significant adjusted *p*-value of 1.17 × 10^−6^ (*p* ≤ 0.05) across all matrices. However, multiple comparisons among the assessed matrices using the Tukey HSD post-hoc revealed that only significant differences between soil-irrigation water (*p* = 5.54 × 10^−5^), soil-nearby water (*p* = 4.21 × 10^−5^), manure-irrigation water (*p* = 8.62 × 10^−3^) and manure-nearby water (*p* = 7.04 × 10^−3^). Soil-manure (*p* = 0.55) and irrigation-water-nearby freshwater (*p* = 0.99) combinations were not statistically different.

As shown in Figure 4A, the soil matrix appeared to have the highest diversity with 81 different serovars. Of these serovars, the most dominant serovars were *Salmonella enterica* subsp. *enterica* serovar Heidelberg with 17%, followed by 11% of *S. enterica* subsp. *enterica* serovar Enteritidis, 8% Newport, 7% Agona, 4% Typhimurium, 3% Montevideo and 1%-2% of other serovars. The 81 *Salmonella* spp. consisted of *Salmonella bongori, S. enterica* subsp. *arizonae*, *S. enterica* subsp. *diarizonae, S.* Adelaide, Agona, Alachua, Anatum, Baildon, Bareilly, Berta, Blegdam, Bovismorbificans, Braenderup, Cerro, Choleraesuis, Cubana, Derby, Dublin, Eastbourne, Enteritidis, Gallinarum, Gallinarum, Gaminara, Give, Hadar, Hartford, Havana, Heidelberg, Houtenae, Hvittingfoss, Indica, Infantis, Inverness, Java, Javiana Johannesburg, Kentucky, London, Manhattan, Mbandaka, Meleagridis, Minnesota, Mississippi, Montevideo, Moscow, Muenchen, Muenster, Nchanga, Newport, Nitra, Norwich, Ohio, Oranienburg, Paratyphi A, Paratyphi B, Paratyphi C, Pullorum, Rissen, Rough, Rubislaw, Saintpaul, Schwarzengrund, Salamae, Senftenberg, Soerenga, Stanley Tallahassee, Tennessee, Typhi, Typhimurium, Uganda, Urbana, Virchow, Wandsworth, Weltevreden, Weslaco and Worthington.

For manure samples (Figure 4B), the most dominant strain was also *S.* Heidelberg with 18%, followed by 11% Enteritidis, 10% Newport, 5% Agona, 4% Typhimurium, 3% Senftenberg and 1%–2% of other serovars. In total, 68 serovars were found in manure samples. These strains included *S. bongori, S. enterica* subsp. *arizonae*, *diarizonae*, *enterica*, *S. enterica* subsp. serovars Adelaide, Agona, Alachua, Anatum, Apapa, Aqua, Baildon, Bareilly, Bovismorbificans, Braenderup, Cerro, Choleraesuis, Cubana, Derby, Dublin, Eastbourne, Enteritidis, Gallinarum, Gaminara, Give, Havana, Heidelberg, Houtenae, Infantis, Inverness, Johannesburg, Kentucky, London, Manhattan, Mele, Milwaukee, Minnesota, Mississippi, Montevideo, Muenchen, Nchanga, Newport, Norwich, Oranienburg, Paratyphi, Paratyphi A, Paratyphi B, Paratyphi C, Pullorum, Risse, Rubislaw, Saintpaul, Salamae, Senftenberg, Stanley, Tallahassee, Tennessee, Thampson, Typhi, Typhimurium, Uganda, Urbana, Virchow, Wandsworth, Weltevreden and Worthington.

Unlike soil and manure samples, irrigation water had low diversity (Figure 4C), with *S.* Typhimurium being the most abundant (17%), followed by 11% of *S.* Johannesburg, *S.* Heidelburg, *S. enterica* subsp. *enterica*. In addition, the following species and subspecies accounted for 5% to 6% of the diversity of irrigation samples: *Salmonella enterica, Salmonella enterica* subsp. *enterica, Salmonella enterica* subsp. *enterica* serovars Typhimurium, Johannesburg, Heidelberg, Newport, Inverness, Gallinarum, Choleraesuis, Cerro, Bovismorbificans and Alachua.

Similar to irrigation water, freshwater samples had low *Salmonella* diversity (Figure 4D), of which the most abundant was *Salmonella enterica* subsp. *enterica* (19%), followed by *Salmonella enterica* (18%) and other isolates serovars accounted for 9% of the total population. The following species and subspecies were found: *S. bongori*, *S. enterica* subsp. *enterica* serovars Weltevreden, Enteritidis, Typhimurium, Typhi, Heidelberg and Bovismorbificans.

**Figure 5:**
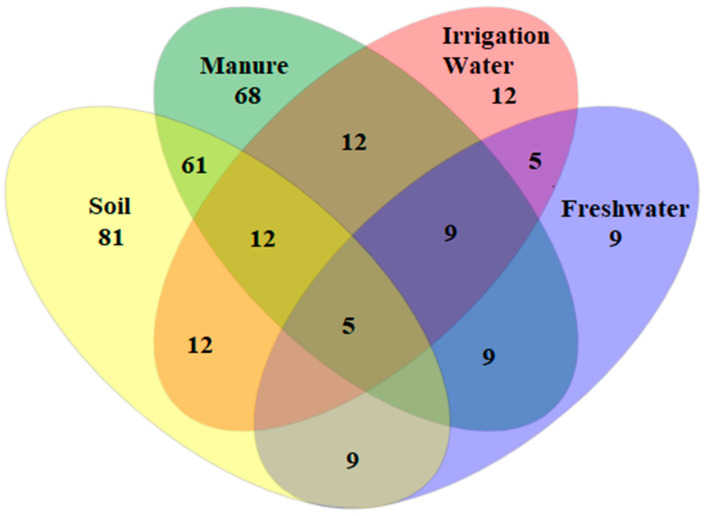
A Venn diagram depicts the overlap (or shared) ESBL-producing *Salmonella* spp. serovars in a multidimensional network between soil, manure, irrigation water and freshwater matrices. The number inside overlapping ellipses denotes shared serovars.

To assess the shared ESBL-producing *Salmonella* spp. serovars across all sampled matrices, a Venn diagram was built (Figure 5). Numbers inside shared intersecting circles, which represent the number of shared serovars in soil, manure, irrigation water and freshwater stream environment. The four environments shared five serovars. Twelve serovars were shared between soil and manure. Irrigation and freshwater shared five serovars.

## 4. Discussion

Worldwide, there has been an increase in reports of ESBLs-producing *Salmonella* spp., including Central Europe [41], South, Eastern and Western Asia [42], North America [43] and South and North Africa [44]. Although the presence of ESBL-producing *Salmonella* spp. has become a great public health concern worldwide [45], studies linking environment to the clinical occurrence of ESBL-producing *Salmonella* are still few, especially in South Africa. It is, therefore, important to track the prevalence, dissemination and diversity of *Salmonella* from agricultural environments to the aquatic environment, which is used in the developing world for drinking and recreational purposes. Thus, this study was conducted to uncover the diversity and related strains of ESBL-producing *Salmonella* between agricultural settings (soil, manure and irrigation water) and a nearby freshwater stream. Due to various limitations associated with culture-based methods [46,47,48], in this study, we employed both culture-dependent and independent methods to investigate the dissemination of ESBL-producing *Salmonella* spp. from agricultural to aquatic environments. 

Using CHROMagar™ Salmonella Plus media and CHROMagar™ Salmonella Plus media supplemented with CHROMagar™ ESBL Supplement, results of the present study revealed the prevalence of *Salmonella* spp. and ESBL-producing *Salmonella* spp. from both agricultural matrixes (soil, manure, irrigation water) and freshwater stream samples. The highest prevalence of *Salmonella* spp. (62.5%, 65/104) and ESBL-producing *Salmonella* (34.6%, 36/104) isolates were detected in soil samples as compared to other sample matrices. This might be due to factors such as temperature, moisture, soil type, UV light and soil organisms that contribute to the survival of *Salmonella* [49] or the fact that the soil acts as a recipient of all contaminated sources during the farming process. Although the manure applied for soil fertility and the stored-dam water used for irrigation were also found to be contaminated with both *Salmonella* spp. and ESBL-producing *Salmonella*, a gradual decrease in the prevalence of these organisms was observed from soil to nearby water sources (Figure 1). Our findings corroborate those of Adzitey et al. [50], who also highlighted that only 12 out of 275 different drinking water samples tested positive for *Salmonella* spp. Furthermore, the findings of the present study revealed that the prevalence of *Salmonella* spp. was significantly higher than that of ESBL-producing *Salmonella* in all four matrices (1.80 to 3.13 fold) during the sampling regime (from June to September 2018). These findings also agree with previous investigators who reported that the prevalence of ESBL-producing *Salmonella* varied in different provinces, sampling years and sampling seasons [51]. 

During the study period, MALDI-TOF Biotyper was used for the identification of *Salmonella* spp. and ESBL-producing *Salmonella* spp. isolates, as it provides high accuracy in species-level identification [52]. Previous investigators have also used this method to identify and discriminate *Salmonella* spp. from other species. Out of 147 isolates obtained from all matrices, 134 (91.2%) isolates were identified as *Salmonella* spp. In addition to this bacterium, other species such as *E. coli, Pseudomonas putida, Pseudomonas fulva and Stenotrophomonas maltophilia* (*Pseudomonas hibiscicola*) were also identified. The detection of these species might be due to environmental complexity and the fitness of the environment. Except for Vi-antigen, all the serogroups (A, B, C, D, E or G) were found in ESBL-producing *Salmonella* spp. isolated from soil, with the serogroups C reflecting as the highest serogroup (*n* = 21) and E or G as the lower (*n* = 1). Although manure isolates harboured four serogroups (B, C, D, E or G), with serogroup C (*n* = 18) being the most identified, no serogroups with A and Vi-antigen were found. Despite its predominance in all isolates of the matrixes in this study, serogroup C was not found in irrigation water isolates.

Nevertheless, one isolate from this type of water exceptionally harboured the Vi-antigen that was not identified in other matrix isolates. In this study, freshwater harboured only three types of serogroups (A, C and D). Overall, the findings of this study showed that ESBL-producing *Salmonella* isolates were successfully serogrouped, and the majority of isolates belonged to serogroup C. A previous study that focused on poultry isolates also reported that 97% of the isolates belonged to serogroups B, C1 and C2 [53]. Roy et al. [54] also found that the majority of poultry (95%) harboured *Salmonella* isolates, which belonged to serogroups B and C. These findings have shown that serogroup C remains the most predominant in agricultural settings. Our findings are in agreement with previous studies, which revealed serogroup C as the most prevalent serogroup and suggested that strains within this serogroup are multidrug-resistant, as reported elsewhere [55,56,57]. 

To assess the genetic attributes behind the observed resistivity, molecular characterisation was done, and the presence of resistant genes such as *bla*_OXA_, *bla*_TEM_, *bla*_SHV_, *bla*_CTX-M_ and one gene that encodes for the resistance to sulphonamides (*sul*1) were ascertained. Overall, *bla*_OXA_ gene was found to be the most commonly detected ARG (75%) in all ESBL-producing *Salmonella* spp. isolates from all the matrices, while no isolates carried *bla*_SHV_. It was also noted that the soil isolates did not harbour *bla*_TEM_ (Figure 2). Previous studies have implicated genes such as *bla*_TEM_ and *bla*_CTX-M_ as responsible for the appearance of resistance to third-generation cephalosporins [58,59].

Moreover, the *sul*1 gene has been detected in most *Salmonella* isolates that exhibit resistance to trimethoprim-sulfamethoxazole and *bla*_TEM,_ and *bla*_OXA_ genes have been described as the enzymes most frequently related to ampicillin and amoxicillin/clavulanate resistance [60]. Binh et al. [61] detected the abundance of the resistance genes *sul*1, *sul*2 and *bla*_TEM_ in field-scale manures. Another study reported *sul*1 as the predominant gene in *S.* Typhimurium [62]. Soil isolates have been shown to have a high diversity of ARG. Our findings were found to be similar to Durso et al. [63] and Nesme et al. [64], where metagenomic data analyses suggest that the soil has a high diversity of ARG. The fact that manure and irrigation water are used in agriculture to enhance the fertility of the soil and the growth of crops means these factors could influence the diversity of ARG in soil. The *bla*_TEM_ was consistently present in soil, irrigation water and freshwater samples, but not in the manure samples. The first report of the occurrence of the *bla*_TEM_ gene in *Salmonella* recovered from animal faeces was identified in the Eastern Cape province of South Africa [65]. It should be mentioned that the presence of antibiotics, antibiotic-resistant bacteria (ARB) and ARG have been detected in numerous rivers worldwide [66]. Among the genes, β-lactamases were also reported, e.g., *bla*_CTX−M_, *bla*_IMP_, *bla*_VIM_, *bla*_KPC_ and variants of these genes may encode ESBL or carbapenemase activity [67]. In this study, a low number of ARG were noted in freshwater stream samples. According to Foote et al. [68], low concentrations of ARG and ARB in large freshwater streams might be due to the water current and strong tide. The contamination of ESBL-positive bacteria and associated genes (*bla*_SHV_, *bla*_CTX-M-15_ and *bla*_TEM_) have also been observed in environmental and drinking water sources in Nigeria [69]. Since rivers and lakes are used as a source of irrigational and recreational purposes [70], the presence of these ARG in water bodies represents a public health concern. Other study revealed the presence of *bla*_TEM-1_ and *bla*_CTX-M_ genes in the same genetic environment in clinical *Enterobacteriaceae* isolates, producing *bla*_CTX-M-1_ type β-lactamases [71].

In this study, we also assessed virulence genes such as *spiC, pipD, spaM, orfL* and *misL* genes. The *orfL* and *misL* genes have been reported to be responsible for the survival of *Salmonella* in host macrophages. The *orfL* gene is also involved in adhesion, autotransportation and colonisation and is found in SPI-4 [72]. The *pipD* gene is a type III secreted effector associated with the SPI-1 system and is found in SPI-5 [72]. When assessing the virulence profiles of isolated ESBL-producing *Salmonella* spp., the most commonly detected VF was *orfL* in 40.0% (16/40) of ESBL-producing *Salmonella* spp. This VF was followed by *misL* in 32.5% (13/40)_,_ *pipD* in 30.0% (12/40), *spaM* in 25.0% (10/40) and the lowest was spiC in 17.5% (7/40). Our findings differ from those of Zishiri et al. [27], who reported the virulence genes isolated from South African clinic isolates that harboured 85% of *spi*C gene, followed by the *pip*D (80%), then *mis*L (75%) and finally 20% of *orf*L genes. The reason might be due to the fact that our isolates were recovered from agricultural environments. It should be noted that the presence of the virulence gene in the majority of agricultural isolates highlights the role of this virulence gene in the production of enterotoxin, which is responsible for causing acute gastroenteritis. Thus, the study of virulence genes spreading in different *Salmonella* isolates would contribute to a better understanding of *Salmonella* pathogenicity. 

The presence of the VF described above was frequently associated with the *inv*A gene. This gene is found in SPI-1 and is vital because it is conserved in all *Salmonella* and is well-known as invasion gene A (*invA*), which is responsible for host invasion [73]. Although reported not to be present in all *Salmonella* spp., it has been established by the U.S. Food and Drug Administration as a confirmatory gene for pathogenic *Salmonella* spp. [74]. Furthermore, as highlighted above, it possesses the ability to reveal up to 86 serovars [31]. However, in this study, all suspected ESBL-producing *Salmonella* spp. isolates grown on ESBL-supplemented CHROMagar™ Salmonella Plus plates were positive for the *invA* gene. These findings are in agreement with a previous study conducted by Arafat et al. [75], which showed that all isolates possessed the *invA* gene. However, results from Kadry et al. [30] revealed that in eight *Salmonella* isolates, only 50% were positive for *invA* gene in both egg (*S*. Typhimurium) and human (*S*. Virchow and *S*. Kentucky) isolates. To the best of our knowledge, there is a paucity of studies revealing the VF harboured in *Salmonella* spp. isolated from agricultural environments. 

In this study, we found 81, 68, 12 and 9 ESBL-producing *Salmonella* spp. serovars from soil, manure, irrigation water and nearby freshwater, respectively. Soil was shown to have an abundant number of serovars as compared to other matrices. We found that five *Salmonella* spp. serovars were shared among soil, manure, irrigation water and nearby water sources. Interestingly, soil and manure shared more *Salmonella* spp. serovars (*n* = 61), while irrigation water and freshwater (*n* = 5) and soil and irrigation water (*n* = 12) shared the same number of serovars (Figure 5). However, there were 19 and 7 unique ESBL-resistant *Salmonella* serovars found in soil and manure, respectively. Notably, all irrigation water and freshwater *Salmonella* serovars were found in both soil and manure samples. One explanation for the lower occurrence of ESBL-producing *Salmonella* spp. in irrigation water and freshwater might be to the matrix physical state as microorganisms tend to be unevenly distributed, unlike in a solid matrix. The presence of all certain serovars from soil and manure samples found in freshwater might be due to agricultural runoff during heavy rains. In contrast, the presence of all irrigation water *Salmonella* spp. in soil could indicate how untreated wastewater used as irrigation water might pollute the receiving soil. 

Overall, we found two species of *Salmonella,* which are *S. enterica* and *S. bongori* with the diverse *S. enterica* subspecies and serovars (Figure 4). In the United States and Canada, *S.* Heidelberg was frequently isolated from clinical salmonellosis cases, retail meats and livestock serotype [76]. These results were similar to our findings, where the most abundant serovar was *S.* Heidelberg. However, from 1996 to 2006, South Africa veterinary diagnostic laboratory data revealed that the most common *Salmonella* serovars were *S.* Typhimurium, Enteritidis, Isangi, Infantis, Dublin, Heidelberg, Virchow, Newport, Muenchen, Hadar, Anatum, Arizonae and Schwarzengrund [77]. *Salmonella* species with various serovars, *S.* Typhi, the highest at 69/119 (57.9%), followed by serovar Typhimurium at 28/119 (23.5%) were serotyped from a tertiary hospital in Eastern Cape, South Africa [78]. Studies have shown the presence of a large diversity of different serovars in the aquatic environments [79]. In Ouagadougou, 22 different serotypes were isolated from surface water [80]. These serovars imply that other external factors may also play an important role in AR dissemination.

## 5. Conclusions

To the best of our knowledge, this study represents one of the few reports investigating the dissemination of ESBL-producing *Salmonella* spp. from agricultural to aquatic environments using a Pretoria North farm as a case study. This study provides valuable information on the antibiotic resistance, virulence gene content and serovar diversity in ESBL-producing *Salmonella* isolated from soil, manure, irrigation water and freshwater stream samples. The high rate of ESBL-producing *Salmonella* species was revealed, but the molecular investigation also determined the presence of *sul*1 genes associated with virulence factors. The most common resistant gene was *bla*_OXA,_ followed by *bla*_CTX,_ *bla*_TEM_ and lastly *sul*1. *Bla*_SHV_ was not detected in all assessed matrices. Soil was shown to have high diversity and is presented as a major reservoir of ESBL-producing *Salmonella* sp. Our findings conclude that agricultural environment contamination may have a direct relationship with the presence of antibiotic-resistant *Salmonella* in freshwater stream samples. The presence of ESBL-producing *Salmonella* in freshwater stream samples is a potential health risk. To overcome the dissemination of ARB and ARG from agricultural environments, antimicrobial resistance surveillance needs to be implemented in agricultural environments to reduce the dissemination of ESBL-producing *Salmonella* to aquatic environments.

## Figures and Tables

**Table 1:** Oligonucleotide primers in the PCR assay.

Genes	Nucleotide Sequences (5′-3′)	Target Size	*T*_Annealing_ (°C)	Reference
*Salmonella* spp. ARG				
*bla*_CTX_	F:ATGTGCAGYACCAGTAARGTKATGGC R:TGGGTRAARTARGTSACCAGAAYCAGCGG	593	60	[24]
*bla*_SHV_	F:TTCGCCTGTGTATTATCTCCCTG R:TTAGCGTTGCCAGTGYTCG	854	50	[24]
*bla*_OXA-1_	F:ATGAAAAACACAATACATATCAACTTCGC R:GTGTGTTTAGAATGGTGATCGCATT	820	58	[24]
*sul*1	F:GCGCGGCGTGGGCTACCT R:GATTTCCGCGACACCGAGACAA	350	65	[25]
*bla*_TEM_	F:ATGAGTATTCAACATTTCCG R:ACCAATGCTTAATCAGTGAG	859	53	[26]
*Salmonella* spp. VF				
*spi*C	F:CCTGGATAATGACTATTGAT R:AGTTTATGGTGATTGCGTAT	309	54	[27]
*mis*L	F:GTCGGCGAATGCCGCGAATA R:GCGCTGTTAACGCTAATAGT	400	58	[27]
*pip*D	F:CGGCGATTCATGACTTTGAT R:CGTTATCATTCGGATCGTAA	350	56	[27]
*spa*M	F:CGCTGTACGGTATTTCATT R:CTGACTCGGCCTCTTCCTG	394	55	[28]
*orf*L	F:GGAGTATCGATAAAGATGTT R:GCGCGTAACGTCAGAATCAA	550	58	[27]
*inv*A	F:GTGAAATTATCGCCACGTTCGGGCAA R:TCATCGCACCGTCAAAGGAACC	284	45	[28]
